# Improvement of saffron production using *Curtobacterium herbarum* as a bioinoculant under greenhouse conditions

**DOI:** 10.3934/microbiol.2017.3.354

**Published:** 2017-05-22

**Authors:** Alexandra Díez-Méndez, Raul Rivas

**Affiliations:** 1 Department of Microbiology and Genetics, Edificio Departamental de Biología, Universidad de Salamanca, (USAL) Dres. de la Reina s/n, 37007, Salamanca, Spain; 2 Instituto Hispano Luso de Investigaciones Agrarias (CIALE), Salamanca, Spain; 3 Associated Unit USAL-CSIC (IRNASA), Salamanca, Spain

**Keywords:** endophyte, *Crocus sativus*, PGPRs, biofertilizer, agriculture, *Crocus serotinus subs clusii*

## Abstract

Plant Growth Promoting Rhizobacteria (PGPR) are natural soil bacteria which establish a beneficial relationship with their host. This microbiota community exists in the rhizosphere and inside plant tissues and stimulates plant growth by a variety of direct or indirect mechanisms. These bacterial plant promoters are frequently present in different environments, and are associated with many plant species, both wild and agricultural. Saffron is the dried stigmas of *Crocus sativus* (L.) and is the most expensive spice in the world. Remarkably, saffron cultivation and collection is carried out by hand and does not involve the use of machines. Additionally, 150 flowers are needed to produce one gram of dried stigmas. Hence, a slight increase in the size of the saffron filaments per plant would result in a significant increase in the production of this spice. In this study, we report the improved production of saffron using *Curtobacterium herbarum* Cs10, isolated from *Crocus seronitus* subs *clusii*, as a bioinoculant. The bacterial strain was selected owing to its multifunctional ability to produce siderophores, solubilize phosphate and to produce plant growth hormones like IAA. Furthermore, the isolate was tested on saffron producing plants under greenhouse conditions. The results indicate that *Curtobacterium herbarum* Cs10 improves the number of flowers and significantly enhances the length of the saffron filaments and overall saffron production compared to the control treated plants.

## 1. Introduction

The rhizosphere is a hot spot of microbial diversity where root exudates provide energy sources and nutrients, which result in an increase in the microbial population within this ecological niche [1]. Rhizosphere microorganisms can be neutral or pathogenic, whereas other microbes can establish a beneficial relationship with their host, such as those known as plant growth promoting rhizobacteria (PGPR) [2]. There are mainly two types of soil bacteria that can act as PGPR: rhizospheric bacteria, defined as the rhizospheric biota able to colonize root surfaces [3] and endophytic bacteria, defined as the bacteria that colonize plant tissues, which reside in the vascular system [5], in the intercellular space [6] or within the cells [7] without producing any negative damage to the host [4]. Both types of bacteria trigger similar mechanisms of growth stimulation [8],[9]. The effects of PGPR take place through different types of processes, which are classified into two types of mechanisms: indirect or direct [10]. Indirect mechanisms refer to those processes that occur outside the plant and include improved nutrient availability, such as free nitrogen fixation [11],[12],[13], phosphate solubilisation activity [14], the production of an iron chelation siderophore [15], and also those processes involved in the biocontrol of microbial pathogens [16]. Direct mechanisms on the other hand, occur inside the plant and directly affect to host's metabolism, such as in the production of phytohormones (auxins, cytokinins and gibberellins) [17], and/or the reduction of abiotic stress, such as plant ethylene levels by synthesising ACC-deaminase [18].

These bacterial plant promoters are commonly present in different environments and are associated with many plant species, both wild and agricultural [9]. Examples of these types of plants include wild rice species [19], *Pelargonium graveolens* [20], *Rhizophora mangle* [21], *Laguncularia mangroove* [22], forest species like oak [23], and cactus species like *Manillaria carnea* [24]. Also, bacterial plant promoters have been described in different crops like *Phaseolus vulgaris* [25], *Solanum tuberosum* [26], *Beta vulgaris* [27], *Zea mays* [28] and oat [29]. Despite the diverse relationships that can occur between bacteria and plants, there are few data concerning agriculture species, including the saffron producing plant *Crocus sativus* (L.); although recently, the isolation of different rhizobacteria strains from the rhizosphere of *Crocus sativus* (L.), such as *Pseudomonas sp, Klebsiella sp, Bacillus subtilis, Acinetobacter haemolyticus, Acinetobacter lwofii, Pantoea sp*, [30] has been reported.

Saffron is comprised of the dried stigmas of *Crocus sativus* (L.), which belongs to the Iridaceae family, and is the most expensive spice in the world [31]. Saffron is mainly used as a flavouring agent and as a food colourant [32]–[35], and Iran, India and Spain are the largest producers [36] of this spice. Remarkably, saffron cultivation and collection is done solely by hand, without the use of machines, and 150 flowers are needed to produce one gram of dried stigmas. Hence, a slight increase in the size of the saffron filaments per plant would result in a significant increase in its overall production. In addition, there are only a few published reports regarding the effects of plant promoters on the production of saffron, finding data about it, after using the register biofertilizers *Bacillus subtilis* FZB24 [31]. Thus, the aim of this study was to evaluate the use of the endophyte *Curtobacterium herbarum* Cs10, isolated from wild *Crocus serotinus* subs *clusii*, as a potential bioinoculant in saffron production.

## 2. Materials and Method

### 2.1. Bacterial strain used in this study

The strain Cs10 was isolated from a *Crocus serotinus* subs *clusii* bulb. The bulb was surface sterilised in a Cl_2_Hg (2.5%) solution for 10 minutes followed by five washes in sterilised H_2_O. Then, the bulb was crushed and plated on YEDP medium (Glucose 7 g/L; Bacto Yeast Extract 3 g/L; Calcium hydrogen phosphate anhydrous 3 g/L and agar 15 g/L) under sterile conditions. Some bulbs were cultured in the appropriate medium and used as negative controls. One colony was re-streaked on YEDP medium plates until a pure culture was obtained.

### 2.2. DNA extraction, PCR amplification of the 16SrRNA gene and sequencing

DNA was extracted from the pure culture using the REDExtract-n-Amp™ Plant PCR Kit (Sigma) following the manufacturer's instructions. PCR was performed using the 27F (5′AGAGTTTGATCCTGGCTCAG) and 1522R (5′AAGGAGGTGATCCNCCRCA) bacteria-specific primers under the following conditions: preheating at 95 °C for 9 minutes, 35 cycles of denaturation at 95 °C for 1 min, annealing at 54 °C for 1 minute and extension at 72 °C during 1 minute, and a final extension at 72 °C for 7 minutes. The PCR amplicon was run on a 1% agarose (w/v) gel and the size was checked using the GeneRuler 1 Kb DNA Ladder (Thermo Scientific™). The DNA fragments were isolated and purified using the GeneJet Gel Extraction and DNA Cleanup Micro Kit (Thermo Scientific™) according to the manufacturer's instructions. The fragment was sequenced at the Macrogen DNA Sequencing Service (Macrogen, Europe). The sequences of both strands were matched using a global alignment and a contig was created using the CAP contig assembly program of BioEdit ver 7.2.5. The consensus sequence was submitted to EZBiocloud at the website http://www.ezbiocloud.net.

### 2.3. Phylogenetic analysis

The sequences that were the closest were selected and aligned using the multiple alignment program Muscle of MEGA7. A distance matrix was generated and a phylogenetic tree was constructed using a Neighbour-joining algorithm [37]. The reliability of the phylogenetic tree generated from the above analyses was assessed by using a bootstrap program in sets of 1000 re-samplings (MEGA 7).

### 2.4. In vitro PGPR culture-dependent methods

Phosphate solubilisation was tested in YED-P medium [38] and iron chelation, via siderophores production, was determined by the formation of an orange-yellow halo in M9-CAS-AGAR [39]. Indole acetic acid (IAA) production was evaluated in JMM medium supplemented with 107 mg l^−1^ of tryptophan [40]. After 5 days of incubation at 28 °C, the culture supernatants were recovered by centrifugation and subsequently filtered. Analysis of IAA was carried out in the Elemental Analysis, Chromatography and Mass Spectrometry Service of NUCLEUS (University of Salamanca) in an Agilent 1100 HPLC, using a trap XCT ion trap mass spectrometer as the MS/MS detector. An ascent is express C18 column 10 cm length and 2.1 mm diameter with 2.7-micron particle size was used. Eluent A was water with 0.1% formic acid and eluent B was acetonitrile. The initial conditions were 90% A and 10% B. A gradient was used to reach at 3 min. 20% of B and at 20 min. 90% of B. An IAA D2 internal standard was used in the analysis.

### 2.5. Greenhouse assay

*Crocus sativus* bulbs were sown in non-sterile soil (3:1 peat:vermiculite). A total of 15 plants per treatment were used (1 plant per plot). Thirteen days post saffron sprout, a single inoculation of 50 mL of a Cs10 strain suspension (10^7^ cells mL^−1^) was performed. Posts were watered weekly and no fertilisers were added. The flowers were collected each week. Plants were harvested 3 weeks after inoculation and the total number of flowers, the amount of saffron and the length of the saffron threads were determined.

### 2.6. Statistical analyses

Data were statistically analysed using the one-way analysis of variance (one-way ANOVA) technique. The mean values were compared using the Fisher protected LSD test (p = 0.05) and the Statview software 5.0 was used for the statistical analyses.

## 3. Results and Discussion

### 3.1. Bacterial isolation and identification

The use of PGPR is considered as an ecological alternative to chemical fertilisers due to their ability to enhance yield production, reduce abiotic or biotic stress and confer protection against pathogens [41]. Thus, research based on agriculture focuses mainly on the rhizosphere because this environment is rich in microbial diversity [42]. However, the isolation of endophytes has received much attention in the last few decades. In this study, bacterial isolation was carried out on 8 plants of *Crocus serotinus* subs *claussii* collected from Calvarrasa de Arriba (40° 55′ 36″ N, 5° 33′ 20″ O) Salamanca, Spain. As a result, the strain Cs10 was isolated from the bulbs unlike the rest of bacterial strain. This result showed that this type of bulb is not a place where bacteria normally live, which may be due to the fact that plant species belonging to the Iridaceae family synthesise different antimicrobial substances against fungi and gram-positive bacteria [43],[44]. For this reason, and also for its capacity to solubilise phosphate on the isolation medium used, the strain Cs10 was selected and further analysed.

**Figure 1.**
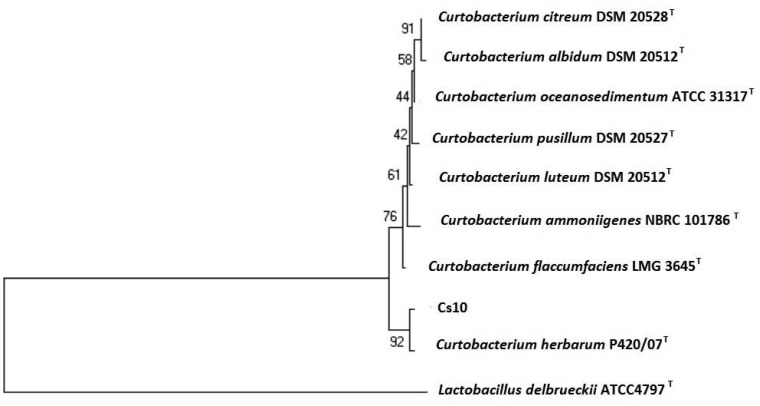
Neighbour-joining tree based on the nearly complete 16S rRNA gene sequences of *Curtobacterium herbarum* Cs10, a species of the genus *Curtobacterium*. The significance each branch is indicated by a percentage bootstrap value calculated from 1000 subsets. Bar, 1 nt substitutions per 100 nt.

To identify bacterial species, we obtained the complete 16S rRNA sequence. The sequence was compared with those from a public database using the FASTA format [45]. It was determined that the isolate belonged to the phylum Actinobacteria, showing 99.8% similarity to the type strain of *Curtobacterium herbarum* (P420/07^T^). A neighbor-joining phylogenetic tree was generated, and the results showed that the closest bacterial species was *Curtobacterium herbarum* (P420/07^T^) (Figure 1). In order for a strain to be considered as a potential PGPR, the microorganism selected must be safe for the environment and for humans [25]. Although some strains belonging to *Curtobacterium flacumfaciens* are well-establish phytopathogens [46], it does not mean that all species from the genera *Curtobacterium* act as pathogens. Bacterial strains belonging to *Curtobacterium ginsengisoli* were isolated from soil in a ginseng field [47] and *Curtobacterium herbarum* was first isolated from the phyllosphere of non-diseased grass [48]. Moreover, the strain Cs10 was isolated from non-diseased *Crocus* *serotinus* subsp. *clusii* plants in this study.

To date, there is no data on native microbes isolated from *Crocus sativus* tissues. However, a recent study reported the isolation of different PGPR belonging to the genera *Pseudomonas, Klebsiella, Bacillus, Acinetobacter* and *Pantoea* within the *Crocus sativus* (L.) rhizosphere [30]; although there are no data regarding their re-inoculation in saffron plants, as well as their impact on saffron production. Despite this, other researchers have inoculated saffron plants with plant promoters isolated from other niches; an example of this is the registered biofertilizer *Bacillus subtilis* FZB24, which increases leaf length, the number of flowers per corm and total stigma biomass [31]. In this study, we selected *Crocus serotinus* subs *clusii* as a microbial source because it is a local plant that lives in soils. Moreover, it belongs to the *Crocus* genus and is catalogued as an autumn flowering as *Crocus sativus* [49]. In addition, both species live in environments characterised by warm dry summers and cool winters [50].

### 3.2. In vitro PGPB mechanism

To evaluate the possible plant growth promoting effects of Cs10, we analysed different culture depend on methods as its ability to synthesise IAA, solubilization of phosphorus and its capacity to chelate siderophore, being all the results positives.

Phosphorus (P) constitutes an important soil nutrient for plant development and its presence in soil can either be from organic or inorganic compounds. The majority of P is highly insoluble and unavailable to plants [51]. Thus, phosphate solubilizing microorganisms play an important role as biofertilizers. Cs10 was able to solubilize phosphate on YEDP medium, as seen by the presence of a degradation halo around the colonies. Diverse associations among agriculture crops like wheat, tomato and radish and phosphate solubilizing bacteria have been reported [3], and this ability can enhance plant growth under certain conditions [52]. Many rhizobacteria are catalogued as microbial phosphate solubilisers such as *Pseudomonas plecoglossicida* (PSB-5) and *Pantoea cypripedii* (PSB-3) [53], different rhizobacteria [54], among others. Therefore, it seems that phosphate solubilisation is a common feature of PGPR, as well as Cs10.

**Figure 2.**
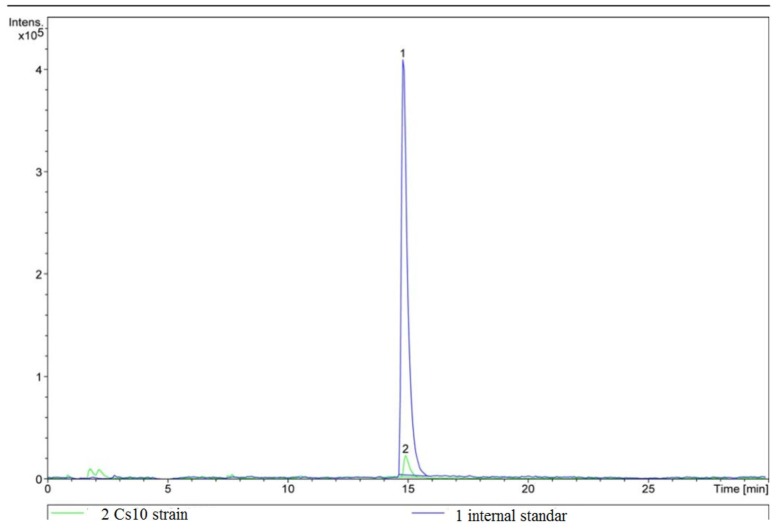
Analysis of IAA. The blue line (1) represents an internal standard; the green line (2) represents the Cs10 strain.

Furthermore, general phytohormones, such as cytokinins, IAA and gibberellins, are produced by plant-associated bacteria and are involved in the improvement of plant growth and reproduction, and help to protect plants against biotic and abiotic factors [55]. Also, IAA is one of the most study phytohormones as it contributes to plant growth and the protection against pathogens [56]. In this study, IAA was measured by HPLC (Figure 2) and a concentration of 11.9 ng/mL was determined. IAA can stimulate plant cell elongation and/or cell proliferation, induce the synthesis of ACC-desaminase, which stimulates the production of ethylene in the plant [18]. Also, this event has been described for different bacterial strains, such as *Burkholderia phytofirmans* PsJN [57], *Enterobacter* sp. 638, *Pseudomonas putida* W619, *Serratia proteamaculans*, [58] which suggests this may be a common plant growth promoting characteristic among rhizobacteria.

Regarding the microbial siderophore, Fe is an essential micronutrient for the survival and proliferation of all plants [59]. Also, it is well known that microorganisms supply Fe to plants, and enhance the growth of the host when the bioavailability of Fe is low [60] and, also is described as an ecological alternative to hazardous pesticides [61]. The bacterial strain Cs10 was surrounded by a yellow-orange halo. Furthermore, bacterial species from the genera *Pseudomonas* and *Streptomyces* are classified as PGPR for their ability to produce siderophores [62],[63].

The results obtained from the mechanisms of the PGPR suggest that theCs10 bacterial strain could be a potential candidate for enhanced saffron production.

### 3.3. Saffron production under greenhouse conditions

PGPR can act as important ecological alternatives that promote plant development by direct or indirect mechanisms. To evaluate the effect of the Cs10 strain with the agronomic saffron, a greenhouse assay was performed. Saffron bulbs were sown in non-sterile soil (3:1 peat: vermiculite) and no chemicals fertilisers were added. As shown in Table 1, the plants treated with the Cs10 strain presented the best results compared with the control treatment. Moreover, we obtained a similar number of flowers in both treatments. However, Cs10 treatment exhibited a significant improvement in the length of the saffron threads (3.10 ± 0.035) and saffron yields (0.013 ± 0.001). These results must be related to the different PGPRs abilities exhibited *in vitro* assays by Cs10 strain. Thus, the *in vitro* abilities shown by Cs10 strain as well as the results already described, indicate that the results obtained in the greenhouse assay were due to a potential synergy among the effects of the PGPR. Both, direct and indirect mechanisms have been described in the literature as promoters of plant development and improved yield production. In agreement with this, our results suggest that the saffron plants treated with the strain Cs10 exhibited longer saffron threads and were heavier than the control plants. All of these improvements may well enhance spice production as there were more flowers per corm and their filaments weighed more.

**Table 1.** Results obtained in the greenhouse assay, including the total number of flowers, the lengths of the saffron threads and saffron yields. A value followed by the same letter in each treatment are not significantly different from each other P = 0.05 according to the Fisher protected LSD test (Least Significant Differences). S.E = Stand Error.* Fifteen plants were included in each treatment. A single greenhouse assay was carried out.

Treatments	Total number of flowers	Saffron threads length (cm) ± S.E*	Saffron yield (g) ± S.E.*
Control	28	2.85 ± 0.0039^a^*	0.007 ± 0.001^a^*
Cs10	29	3.10 ± 0.0035^b^*	0.013 ± 0.001^b^*

To the best of our knowledge, this is the first study regarding the beneficial effects of *Curtobacterium herbarum* Cs10 on saffron production. Due to the PGPR mechanisms exhibited by strain Cs10, we believe it has a potential role as a promoter of saffron growth under greenhouse conditions. Nevertheless, field studies are required.

## 4. Conclusions

In conclusion, our results indicate that *Curtobacterium herbarum* Cs10 can interact with saffron plants and significantly improve the production of this spice. Also, we conclude that *Crocus serotinus* subs *clusii* contains endophytic bacteria that may potentially be used for enhancing saffron production.
